# Genome-wide identification of *HSP90* gene family in *Rosa chinensis* and its response to salt and drought stresses

**DOI:** 10.1007/s13205-024-04052-0

**Published:** 2024-08-18

**Authors:** Jun Xu, Shuangwei Liu, Yueming Ren, Yang You, Zhifang Wang, Yongqiang Zhang, Xinjie Zhu, Ping Hu

**Affiliations:** 1 College of Horticulture and Landscape Architecture, Henan Institute of Science and Technology, Xinxiang, Henan Province China; 2 College of Agricultural, Henan Institute of Science and Technology/Henan International Joint Laboratory of Plant Genetic Improvement and Soil Remediation, Xinxiang, 453003 Henan Province China; 3 Xuchang Academy of Agricultural Sciences, Xuchang, Henan Province China

**Keywords:** *Rosa chinensis*, *HSP90* gene family, Abiotic stress, Relative expression, Protein active site

## Abstract

Heat shock protein 90 (HSP90) is important for many organisms, including plants. Based on the whole genome information, the gene number, gene structure, evolutionary relationship, protein structure, and active site of the HSP90 gene family in *Rosa chinensis* and *Rubus idaeus* were determined, and the expression of the *HSP90* gene under salt, and drought stresses in two rose varieties Wangxifeng and Sweet Avalanche were analyzed. Six and eight *HSP90* genes were identified from *R. chinensis* and *Ru. idaeus*, respectively. Phylogenetic analysis revealed that the analyzed genes were divided into two Groups and four subgroups (Classes 1a, 1b, 2a, and 2b). Although members within the same classes displayed highly similar gene structures, while the gene structures and conserved domains of Group 1 (Class 1a and 1b) and the Group 2 (Class 2a and 2b) are different. Tandem and segmental duplication genes were found in *Ru. idaeus*, but not in *R. chinensis*, perhaps explaining the difference in *HSP90* gene quantity in the two analyzed species. Analysis of cis-acting elements revealed abundant abiotic stress, photolight-response, and hormone-response elements in *R. chinensis* HSP90s. qRT-PCR analysis suggested that *RcHSP90-1–1*, *RcHSP90-5–1* and *RcHSP90-6–1* in Sweet Avalanche and Wangxifeng varieties played important regulatory roles under salt and drought stress. The analysis of protein structure and active sites indicate that the potential different roles of *RcHSP90-1–1*, *RcHSP90-5–1*, and *RcHSP90-6–1* in salt and drought stresses may come from the differences of corresponding protein structures and activation sites. These data will provide information for the breeding of rose varieties with high stress resistance.

**Supplementary Information:**

The online version contains supplementary material available at 10.1007/s13205-024-04052-0.

## Introduction

*Rosa chinensis* belongs to the Rosaceae family. It is one of the most important ornamental plants globally, with significant economic, and cultural value (Omidi et al. 2022). The growth of roses, including *R. chinensis*, is closely related to changes in the external environment. Drought and salt stress limit their growth and outdoor landscape applications, leading to a significant decline in yield and quality (Jia et al. 2021; Su et al. 2023; Geng et al. 2023). Plants are highly sensitive to environmental changes. Changes in environmental conditions such as drought, heat, excess water, and salinity usually affect plant growth and development in a combined manner. Plants adapt to stress by regulating their signaling pathways and changing the expression of related genes (Shang et al. 2013). Therefore, exploring abiotic stress response genes in roses will help to improve their stress tolerance.

Heat shock proteins (HSPs) are chaperones that are present in many prokaryotes and eukaryotes. HSPs are typical stress response proteins. Their gene activity and production can be induced by stimulating environmental parameters such as high temperature, high salt levels, drought, and other stresses, with important biological significance in the adaptation of organisms to stress (Zhang et al. 2013). HSPs are divided into five families based on their molecular weight: sHSP (small molecule heat shock protein), HSP60, HSP70, HSP90, and HSP100 (Wang et al. 2004; Sajad et al. 2022). HSP90 is a heat shock protein family with an approximate molecular weight of 90 kD. It is a homologous dimer chaperone regulated by adenine riboside triphosphate (ATP). HSP90 comprises an N-terminal domain that binds and hydrolyzes ATP, an intermediate domain that recognizes substrate proteins and companion molecules, and a C-terminal domain that participates in dimer formation (Kitaoku et al. 2022).

Known members of the *Hsp90* gene family in model plants including seven in *Arabidopsis* (Krishna & Gloor 2001), 21 in tobacco (Song et al. 2019), seven in tomato (Zai et al. 2015), 10 in poplar (Zhang et al. 2013), six in cucumber (Zhang et al. 2021), 12 in heading cabbage (Sajad et al. 2022), 35 in *Brassica napus* (Wang et al. 2022b), and 10 in pumpkin (Hu et al. 2023b). The *Hsp90* genes of different plants are generally divided into three to five subgroups that play different roles in plant growth. *Hsp90* has important roles in plant seed germination, seedling growth, and the folding, processing, transportation, and degradation of proteins (Reddy et al. 1998; Wang et al. 2004, 2011). *Arabidopsis AtHsp90-1* is strongly expressed in mature seed embryos (Prasinos 2005). *AtHsp90.6* is an essential protein for embryogenesis (Luo et al. 2019). Inhibited production of HSP90s in *Arabidopsis* affects fruiting rate, flowering time, and other characteristics (Sangster & Queitsch 2005).

*Hsp90* is also important in the response of plants to abiotic stress. *HSP90* can control plant growth and development under high-temperature stress by activating the SGT1b TIR1 protein complex (Wang et al. 2016). *HSP90* plays an important role in the YODA cascade that mediates the heat stress response and regulation of stomatal formation to adapt to high-temperature environments (Samakovli et al. 2020a, b; Samakovli et al. 2020a, b). *Arabidopsis* HSP90.1 can activate the substrate protein ZTL, thereby reducing insoluble protein aggregation, refolding erroneous proteins, and improving plant heat tolerance (Gil et al. 2017); HSP90.1 interacts with ROF1 and NBR1 to enhance heat tolerance through heat stress memory (Meiri & Breiman 2009; Thirumalaikumar et al. 2021). Heat stress results in the significantly upregulated expression of *CmoHSP90-3* and *CmoHSP90-6* in pumpkin, while the expression levels of *CmoHSP90-6* and *CmoHSP90-10* are significantly upregulated by cold stress (Hu et al. 2023b). The overexpression of five *GmHSP90s* genes in *Arabidopsis* can increase proline content and enhance the resistance of the plants to abiotic stresses, such as high temperature and drought (Xu et al. 2013). Overexpression of *AtHsp90.2*, *AtHsp90.5*, and *AtHsp90.7* can enhance drought resistance in *Arabidopsis* (Song et al. 2009). In tobacco, *NtHSP90-4*, *NtHSP90-5*, and *NtHSP90-9* are upregulated in response to induction by abscisic acid (ABA), and drought, salt, cold, and heat stress, while *NtHSP90-6* and *NtHSP90-7* are not induced by these conditions (Song et al. 2019). MeHSP90.9 can recruit MeWRKY20 and MeCatalase1 proteins to regulate the drought resistance of cassava (Wei et al. 2020). In soybeans, plants transformed with 12 *GmHSP90s* are strongly induced by high-temperature treatment and exhibit stronger heat and drought resistance (Xu et al. 2013). Finally, in terms of plant immunity, wheat plants overexpressing *TaHsp90.2* and *TaHsp90.3* exhibit significant resistance to stripe rust (Wang et al. 2011). In rice, a companion complex composed of cytoplasmic Hsp90 and its copartner Hop/Sti1 is involved in chitin response and antifungal immunity (Chen et al. 2010).

In-depth research on the function of plant *HSP90* genes under stress is important to understanding plant stress signal transduction and multiple stress response mechanisms, and for improving plant stress resistance. The abiotic stress response mechanisms and expression patterns of the *HSP90* gene family in ornamental plants are still unclear, with few studies performed on the *HSP90* gene in rose plants. In our study, bioinformatics methods were used to identify and analyze the *HSP90* family members of rose. The findings provide a foundation for further studies on the functions of the *HSP90* gene and a theoretical reference for studies seeking to breed more stress-resistant rose varieties.

## Materials and methods

### Plant materials and treatments

'Wangxifeng' and 'Sweet Avalanche' hybrid potted rose varieties were purchased from AIBIDA Horticultural Technology Co., Ltd. (Yunnan, China). These two rose hybrids are the main varieties sold in the market, and the leaves of Wangxifeng are leathery, and the leaves of Sweet Avalanche are paper.

After removing the soil from the selected potted roses that exhibit normal and consistent growth were individually placed in a vase filled with deionized water (Table 1 and Figure S1), and cultivated at 22 ± 1 °C and 60% relative humidity using cycles of 16 h light/8 h dark. Refer to Geng (Geng et al. 2022) and Su's (Su et al. 2023) methods, the experiment adopts a single-factor randomized block design, salt and drought stresses were simulated with 200 mM NaCl and 10% PEG-6000, respectively, for 72 h. Deionized water was used as a control for the same length of time. Leaves were collected taken at 0, 3, 6, 12, 24, 48, and 72 h. The third leaf at the top of each treatment rose was quick-frozen in liquid nitrogen and stored at -80℃, and RNA was extracted for further experiments. Each treatment had three biological replicates.

**Table 1:** Experimental design

Variety	Treatments	Sample time
Wangxifeng	200 mM NaCl	0, 3, 6, 12, 24, 48, and 72 h
	10% PEG-6000	0, 3, 6, 12, 24, 48, and 72 h
Sweet Avalanche	200 mM NaCl	0, 3, 6, 12, 24, 48, and 72 h
	10% PEG-6000	0, 3, 6, 12, 24, 48, and 72 h

### Quantitative real-time reverse transcription polymerase chain reaction (qRT-PCR)

Total RNA was extracted with an RNA Easy Plant Tissue Kit (DP452, Tiangen, Beijing, China). Genomic DNA contaminants were removed by digestion with RNase-free DNase I as described RNA Easy Plant Tissue Kit. RNA concentration and integrity were measured using Nanodrop 2000(Thermo Fisher Scientific Inc, USA). Reverse transcription of RNAs was performed using the HiScript®II 1st Strand cDNA Synthesis Kit (+ gDNA wiper) (Vazyme Biochemical Technology Co., Ltd., Nanjing, China). The cDNA was stored at -20℃. The relative expression level of the target gene was analyzed using qRT-PCR. The SYBR Green detection kit and AceQ qPCR SYBR Green Master Mix (Vazyme, Nanjing, China) were used in the experiments with the QuantStudio6 system (ABI, Waltham, MA, USA) using the rose gene *RcTCTP* (Remay et al. 2009) as the internal control. The program comprised 5 min at 95 °C, then 40 cycles at 95 °C for 10 s and 60 °C for 20 s. The comparative 2^–ΔΔCT^ method was used to calculate relative gene expression (Hu et al. 2023a). All the primers used in the present study (Table S1) were synthesized by Gene Create (Wuhan, China).

### Identification of HSP90 family members

Genome sequences of *R. chinensis*, *Arabidopsis thaliana*, and *Oryza sativa* were downloaded from Ensemble Plants (http://plants.ensembl.org/index.html). The whole genome sequence of *Ru. idaeus* was downloaded from the Genome Database for Rosaceae (https://www.rosaceae.org/). The typical HSP90 domain (PF00183) was downloaded from InterPro (https://www.ebi.ac.uk/interpro/) as the search model. As previously described (Xu et al. 2021), a new hidden Markov model was developed to ensure accurate search results, using hmmbuild (*E*-value < 1 × 10^−5^) in the HMMERv3 suite to search for the HSP90 gene in the whole genomes of rose, Arabidopsis, rice, and raspberry. The Simple Molecular Structure Research Tool (SMART) (http://smart.embl-heidelberg.de/) (Letunic et al. 2021) and National Center for Biotechnology Information-Conserved Domains Database (NCBI-CDD) (https://www.ncbi.nlm.nih.gov/Structure/cdd/wrpsb.cgi) (Lu et al. 2020) were used to examine candidate sequences, reserving sequences containing the complete HSP90 domain for further analysis (Table S2).

### Analysis of physical and chemical properties, phylogeny, gene structure, and conserved motifs

RcHSP90s were submitted to Expasy (https://web.expasy.org/compute_pi/) for the physical and chemical properties prediction protein sequences, and to Plant-mPLoc (http://www.csbio.sjtu.edu.cn/bioinf/plant-multi/) for subcellular localization prediction. A phylogenetic tree (1000 bootstraps) was constructed using the maximum likelihood method of MEGA X software (Kumar et al. 2018) and visualized with EvolView (He et al. 2016). The GFF3 file of roses was used for exon–intron structure analysis. The MEME program (http://memesuite.org/tools/meme) was used for motif analysis. The maximum number of motifs was 20 and the optimal width of each motif was 6–50 residues (Xu et al. 2021). TBtools was used for visualization analysis (Chen et al. 2020).

### Macrocollinearity, gene duplication and *cis*-acting element analyses

The Multicollinearity Scanning Toolkit (MCScanX) was used to identify tandem genes and segmental duplications of the HSP90 gene in roses, as well as macrocollinearity between roses and other species (Wang et al. 2012; Xie et al. 2018). The upstream promoter 2000 bp sequence of HSP90 gene family members was extracted from the rose genome using TBtools. Their cis-regulatory elements were predicted by PlantCARE (http://bioinformatics.psb.ugent.be/webtools/plantcare/html/) and visualized by TBtools software (Chen et al. 2020).

### Heatmap of *RcHSP90s* expression based on RNA-seq data

Using the method described by Liu et al. (https://biotec.njau.edu.cn/plantExp/) (Tian et al. 2018; Liu et al. 2023a), the corresponding expression data of RcHSP90s at different stages in flowers, leaves, and roots under ABA, drought, and salt stress treatments were extracted, and used to construct a heatmap using TBtools (Chen et al. 2020).

### HSP90 protein structure, active sites, and functional diversification

Secondary structure analysis of proteins was performed using SPOMA (https://npsa-prabi.ibcp.fr/cgi-bin/npsa_automat.pl?page=npsa_sopma.html). SPPIDER (http://sppider.cchmc.org/) was used to perform protein active site prediction. As described by Xu et al. (2021), the functional diversification among the subgroups was analyzed using DIVERGE v3.0 software based on the selected protein sequences (Gu et al. 2013).

## Results

### Identification, phylogenetic and physicochemical analyses of *HSP90* family members

**Fig. 1:**
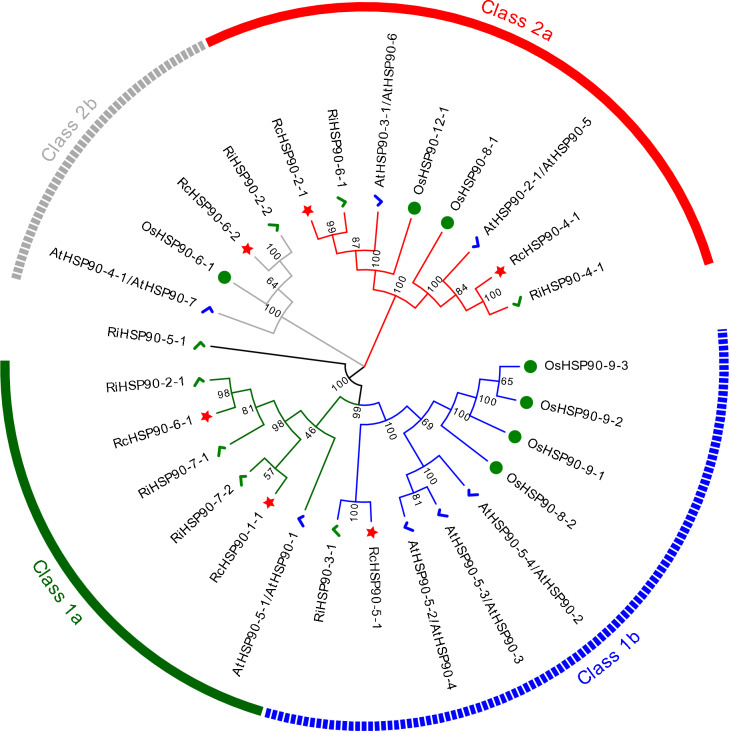
Phylogenetic relationship analysis of 28 HSP90 proteins from *Oryza sativa*, *Arabidopsis thaliana*, *Rosa chinensis*, and *Rubus idaeus*. The diverse classes of HSP90 proteins are marked with different colors. The HSP90 proteins of *Oryza sativa* (Os), *Arabidopsis thaliana* (At), *Rosa chinensis* (Rc), and *Rubus idaeus* (Ri) were represented by green circles, blue checkmarks, red stars, and green checkmarks, respectively. Gene IDs of all the analyzed genes are presented in Table S2

Six, eight, seven, and seven *HSP90* genes were identified from the genomes of rose, raspberry, *Arabidopsis*, and rice, respectively. Members of the *HSP90* family were named according to their gene locations (Table S2). The comparative results of phylogenetic evolutionary trees are shown in Fig. 1. The 28 members were divided into two groups and four subgroups (Classes 1a, 1b, 2a, and 2b). *RiHSP90-5–1* was not included in any subgroup. Among them, Class 1b and 2b have the most and fewest gene members (nine and four, respectively). Among the four subgroups, except for Class 1a, which does not contain rice *HSP90* genes, all other subgroups contain *HSP90* genes from four species. In each subgroup, the *HSP90* members of rose and raspberry, which both belong to the Rosaceae family, cluster together, indicating a closer evolutionary relationship of *HSP90* genes between the two species.

The predicted physicochemical properties of rose HSP90 family member proteins are shown in Table 2. The protein encoded by family members was found to range in length from 447 to 839 amino acids (aa), with a relative molecular weight of 51,719.03 to 94,796.23 D and an isoelectric point of 4.93 to 5.52. RcHSP90-4–1, RiHSP90-2–1, RiHSP90-4–1, and RiHSP90-7–1 are unstable proteins (instability coefficient > 40); all belong to the Class 1a and Class 2a subgroups. The other proteins are stable proteins.

**Table 2:** Information on *HSP90* gene family members

Gene ID	Gene name	Amino acid number	Relative molecular mass/D	pI	Instability index	Subgroup
*RchiOBHm_Chr1g0378391*	*RcHSP90-1–1*	703	80,874.92	4.97	39.35	Class 1a
*RchiOBHm_Chr2g0156661*	*RcHSP90-2–1*	839	94,796.23	5.52	38.99	Class 2a
*RchiOBHm_Chr4g0437121*	*RcHSP90-4–1*	793	89,893.42	4.90	45.19	Class 2a
*RchiOBHm_Chr5g0075541*	*RcHSP90-5–1*	697	80,060.99	4.96	38.49	Class 1b
*RchiOBHm_Chr6g0274441*	*RcHSP90-6–1*	701	80,665.53	4.95	38.98	Class 1a
*RchiOBHm_Chr6g0303071*	*RcHSP90-6–2*	811	92,713.12	4.93	35.96	Class 2b
*Rid.02g056740*	*RiHSP90-2–1*	447	51,719.03	5.14	40.13	Class 1a
*Rid.02g084720*	*RiHSP90-2–2*	819	93,469.93	4.90	35.25	Class 2b
*Rid.03g142450*	*RiHSP90-3–1*	697	80,091.06	4.97	39.32	Class 1b
*Rid.04g153600*	*RiHSP90-4–1*	796	90,265.82	4.88	46.43	Class 2a
*Rid.05g222960*	*RiHSP90-5–1*	704	81,260.94	5.17	34.32	Unclassified
*Rid.06g285060*	*RiHSP90-6–1*	802	91,059.80	5.47	36.02	Class 2a
*Rid.07g301680*	*RiHSP90-7–1*	699	80,400.22	4.97	40.60	Class 1a
*Rid.07g301690*	*RiHSP90-7–2*	698	80,325.32	4.97	38.35	Class 1a

### Chromosomal distribution and collinearity analysis of *HSP90* family members

**Fig. 2:**
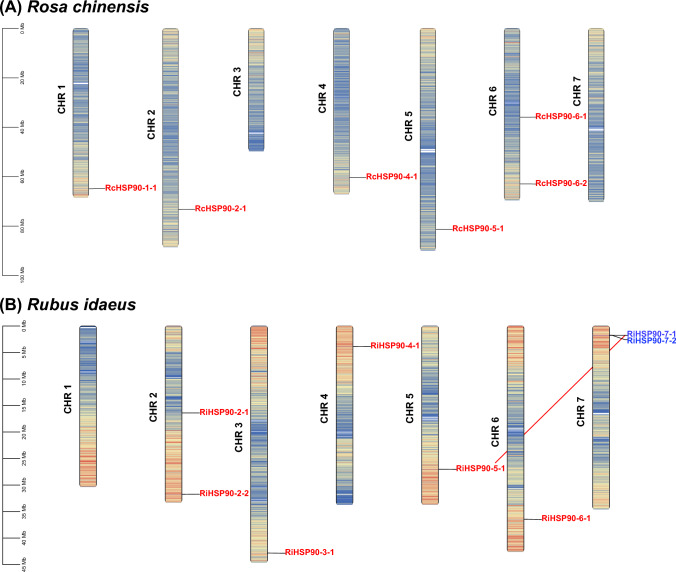
Location, homologous gene pairs, and tandem duplication of HSP90 genes in *Rosa chinensis and Rubus idaeus*. All *HSP90* genes were mapped to their respective loci in *Rosa* chinensis (**A**) and *Rubus idaeus* (**B**). MCScanX software was used to analyze tandem duplication and homologous gene pairs. The genes in blue font indicate genes are generated through tandem repeats, while the genes at both ends of the red line are homologous gene pairs

The distribution of *HSP90* on chromosomes of rose and raspberry is shown in Fig. 2. Six members of *Rosa chinensis* are distributed on five chromosomes and eight members of *Raspberry* are distributed on six chromosomes. One *HSP90* gene was located on chromosome 1, 2, 4, and 5 of roses, and two *HSP90* genes were on chromosome 6. Raspberry has one *HSP90* gene on chromosome 3, 4, 5, and 6, and two *HSP90* genes on chromosome 2 and 7. No tandem genes or segmental duplication genes were presented in the *HSP90* gene of roses. *RiHSP90-5–1* and *RiHSP90-7–1* in raspberry were segmental duplication genes, and *RiHSP90-7–1* and *RiHSP90-7–2* were tandem duplication genes.

**Fig. 3:**
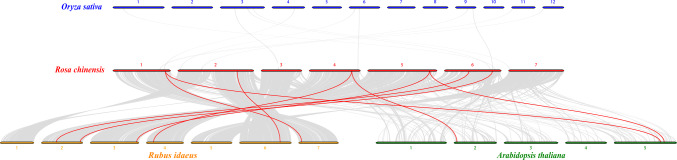
Macro-collinearity analysis of *HSP90* genes between *Oryza sativa*, *Arabidopsis thaliana*, *Rosa chinensis*, and *Rubus idaeus*. Collinearity relationships are shown between two adjacent species. Gray lines in the background indicate the collinear blocks within different genomes and the red lines highlight the syntenic *HSP90* gene pairs

To further explore the relationship between *HSP90* family members among different plants, collinearity maps of roses and rice, roses and *Arabidopsis*, and roses and raspberries were constructed (Fig. 3). The collinearity numbers of *HSP90* family members were zero between roses and rice, three between roses and *Arabidopsis*, and six between roses and raspberries. Compared to rice, roses have more *HSP90* collinearity genes than raspberries and with *Arabidopsis*.

### Analysis of motif sequence and gene structure of *HSP90* family members

**Fig. 4:**
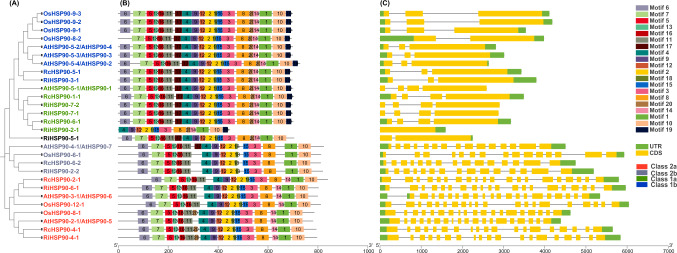
Conserved motifs and gene structure of *HSP90* genes in different species. **A** Phylogenetic tree constructed using the neighbor-joining method with 1000 bootstrap replicates by MEGA X software. **B** Motif composition of HSP90 proteins. **(C)** Exon–intron structure of *HSP90s*. Yellow boxes indicate exons and black lines indicate introns

Motifs of HSP90 genes in rose, raspberry, *Arabidopsis*, and rice were analyzed using MEME software. Results of the analyses of structural characteristics of the family members are shown in Fig. 4. Motifs 1, 2, 3, 4, 8, 9, 10, 12, 15, and 18 were presented in *HSP90* genes in all four plants. Motifs 17 and 19 were presented in Class 1, while Motif 17 (except AtHSP90-4–1) and 19 were absent in Class 2. All HSP90 members except RiHSP90-2–1 contain Motifs 5, 6, 7, 11, 13, and 16. Unlike Class 2b, Class 2a contained Motif 14. The motif composition of members of the *HSP90* gene family in the four plants is similar, with slightly different motif compositions among members of different subgroups. The similarity in motif composition among HSP90 members of the same subgroup is greater.

A significant difference was evident in the number of exons between Classes 1 and Class 2 (Fig. 4C). The number of exons ranges from one to four in Class 1 and from 15 to 20 in Class 2. In Class 1a (except for *RiHSP90-2–1*, which contained one exon) all contained four exons. In Class 1b (except for *OsHSP90-8–2*, which has two exons) all contained three exons. Class 2a contained 19–20 exons and Class 2b contained 15 exons and 14 introns. The similar composition of exons and introns in the same subgroup of the *HSP90* gene in rose samples confirms the conservatism of the HSP90 gene.

### Analysis of *cis* elements in rose *HSP90* family members

**Fig. 5:**
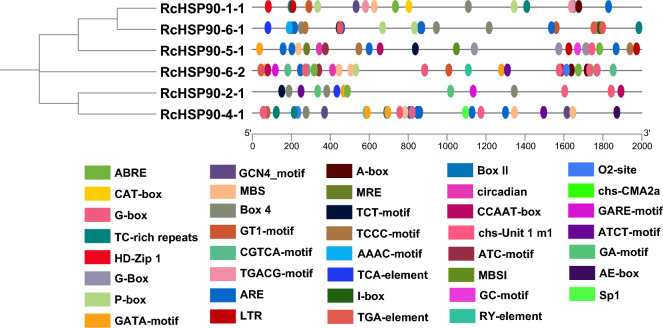
Analysis of cis-acting elements

The upstream sequences of *HSP90* family members contained many non-biological stress elements, photolight-response elements, and hormone-responsive elements(Fig. 5). In the Class 1a subgroup, *RcHSP90-1–1* and *RcHSP90-6–1* possess abiotic stress response elements involved in ABA and gibberellin reactions, as well as defense and low-temperature reactions. In the Class 1b subgroup, *RcHSP90-5–1* has abiotic stress response elements involved in low temperature. In the Class 1b subgroup, *RcHSP90-5–1* has abiotic stress response elements involved in low temperature. In the Class 2a subgroup, *RcHSP90-4–1* has cis-acting elements involved in defense and stress response. Finally, in the Class 2b subgroup, *RcHSP90-6–2* has cis-acting elements involved in low-temperature induction.

### Functional diversification analysis

The functional differentiation analysis findings are shown in Table 3. Among them, the Type-I θ value was 0.1999–0.7810, with a corresponding standard error (SE) of 0.1129. Except for 1b/1a, the p-values of all other subgroups were less than 0.01, indicating a significant difference in their evolutionary rates. Type II analysis revealed θ between 0.0079 and 0.7556, with an SE of 0.2548. Except for 1b/1a, the p-values of all other subgroups were less than 0.01, indicating both a significant level of difference in type II functional differentiation and the presence of functional differentiation caused by amino acid changes in the aforementioned subgroups. The above results indicated that the functional differences between Class 1 and Class 2 genes may due to the changes in evolutionary rate and from changes in certain key amino acid sites. This also applies to the Class 2a and 2b subgroups.

**Table 3:** The results of functional differences between subgroups of type I and type II (**P < 0.01 )

Subgroup	I			II		
	MFE Theta	MFE se	I: P	Theta-II	Theta SE	II: P
1b/ 1a	0.1999	0.1168	0.0782	0.0079	0.0258	0.7598
1b/ 2b	0.7300	0.1356	1.4767E-08**	0.3887	0.0357	0.0000**
1b/ 2a	0.6818	0.1222	2.9318E-09**	0.7556	0.3936	0.0000**
1a/ 2b	0.7377	0.1075	8.9484E-14**	0.2614	0.4085	0.0000**
1a/ 2a	0.7810	0.0977	0.0000**	0.6534	0.4002	0.0000**
2b/ 2a	0.4165	0.0975	4.1939E-06**	0.6711	0.2647	5.3E-07**

### Expression analysis of *RcHSP90s*

**Fig. 6:**
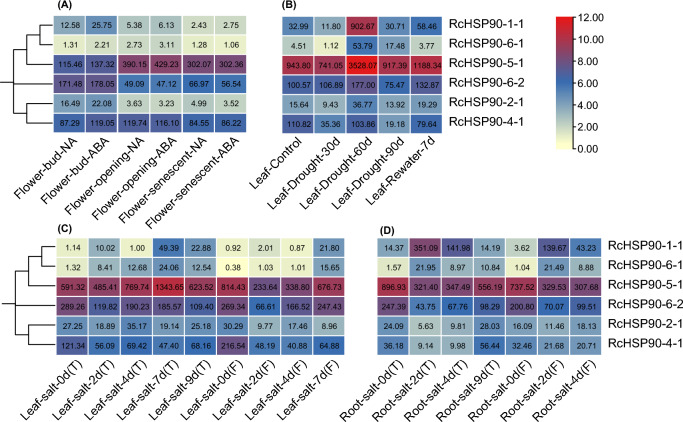
Heatmap of expression profiling of rose *HSP90* genes under different stress. The color scale bar represents the expression values (in log2-based tags per million values) of the genes. The values in square frames represent the tags per million values. T, Salt tolerant varieties; F, Salt sensitive varieties

To elucidate the potential function of *RcHSP90s*, the expression patterns of family members in different organs at different stages and under drought salt stress were assessed. The findings are shown in Fig. 6. The expression level of *RcHSP90s* showed an overall trend of and initial increase followed by a decrease from the bud stage and flowering stage to aging stage (Fig. 6A). Compared with the expression levels in the bud stage without treatment, the expression levels of *RcHSP90-5–1* increased by 237.91% and 161.62% in the flowering and senescence stages, respectively. Upon ABA treatment, the expression levels increased by 212.58% and 120.19%, respectively. Compared with the expression levels during the bud stage without treatment, the expression levels of *RcHSP90-6–2* decreased by 71.37% and 60.95% during the flowering and senescence stages, respectively. Upon ABA treatment, the expression levels decreased by 73.54% and 71.61%, respectively. The expression of *RcHSP90s* in leaves and roots under salt stress is shown in Figs. 6C and D. In salt-tolerant varieties, with increasing salt stress time, the expression level of *RcHSP90-5–1* in leaves first increased and then decreased. In roots the reverse trend was evident. Compared with 0 days, the expression level of *RcHSP90-5–1* in leaves increased by 127.23% at 7 days, while the expression level of *RcHSP90-5–1* in roots decreased by 61.26% at 4 days. Upon imposition of salt stress, expression of *RcHSP90-6–2* first decreased and then increased in both leaves and roots. In salt-sensitive varieties, with increasing salt stress time, the expression levels of *RcHSP90-5–1*, *RcHSP90-6–2*, and *RcHSP90-4–1* in leaves first decreased and then increased. Compared with 0 days, the expression of three genes respectively decreased by 71.31%, 75.27%, and 77.75% at 2 days. With increasing salt stress time, the expression levels of *RcHSP90-5–1*, *RcHSP90-6–2*, and *RcHSP90-4–1* in the roots all decreased. In drought treatment, compared with the control, the expression level of *RcHSP90s* first decreased, then increased, and then decreased with increased of stress time. Compared with the control, the expression levels of *RcHSP90-1–1*, *RcHSP90-6–1*, and *RcHSP90-5–1* in leaves increased by 2636.80%, 1092.68%, and 273.82%, respectively. The collective findings indicate that some members of the *HSP90* family in roses may have positive roles in regulating drought and salt stress.

**Fig. 7:**
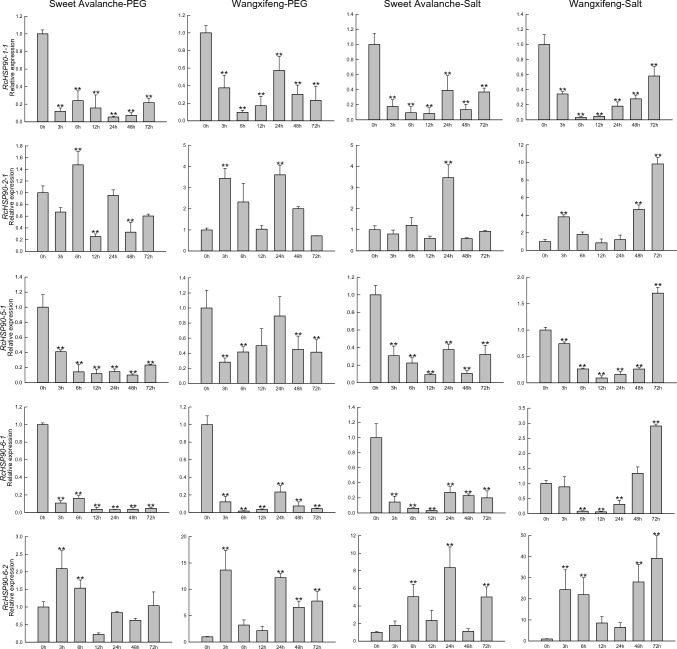
Expression analysis of *RcHSP90s* by qRT-PCR. Error bars indicate the standard error. Asterisks indicate significant differences (assessed using Duncan’s honestly significant difference test), **P < 0.01. All the raw data for qRT-PCR are listed in Table S3

To further investigate the expression changes of *RcHSP90s* under salt and drought stresses, Two different varieties Sweet Avalanche of paper leaves and Wangxifeng of leathery leaves were used for qRT-PCR. The expression status of *HSP90* gene family members under salt and drought stress conditions is shown in Fig. 7. Compared with 0 h, the expression levels of *RcHSP90-1–1* and *RcHSP90-6–1* in the Class 1a subgroup of the two varieties showed a decreasing trend with increasing drought stress time; the difference became significant. Under salt stress, compared with 0 h, the expression levels of *RcHSP90-1–1* in the two varieties also showed a decreasing trend; the difference became significant. However, the expression of *RcHSP90-6–1* differed in the two varieties, with a decreasing trend in Sweet Avalanche that was significantly different. In Wangxifeng, *RcHSP90-6–1* expression first decreased and then increased, with a significant difference. Both *RcHSP90-1–1* and *RcHSP90-6–1* were strongly induced by drought and salt stresses in both Wangxifeng and Sweet Avalanche, indicating that these two genes may actively participate in the adaptive regulation of drought and salt stresses. In the Class 1b subgroup, compared to 0 h, the expression levels of *RcHSP90-5–1* in the two varieties showed an overall decreasing trend under drought and salt stresses, with the difference reaching a significant level. In the Class 2a subgroup, compared to 0 h, *RcHSP90-2–1* expression of the two varieties showed significant differences under salt and drought stresses. The expression of *RcHSP90-6–2* in Class 2b subgroup of Sweet Avalanche was 8.37 times that of 0 h under salt stress, while the expression of *RcHSP90-6–2* in Wangxifeng was 23.80 times that of 0 h under salt stress at 48 h. The expression of *RcHSP90-6–2* in Sweet Avalanche and Wangxifeng increased significantly under drought stress, and the expression of *RcHSP90-6–2* in Wangxifeng at 24 h was 12.23 times that at 0 h under drought stress. This indicates that both Wangxifeng and *RcHSP90-6–2* in Sweet Avalanche were strongly induced under salt stress and participated in the adaptive regulation of salt stress, but *RcHSP90-6–2* in Wangxifeng was more strongly induced. In salt stress, the expression levels of *RcHSP90-1–1*, *RcHSP90-5–1*, and *RcHSP90-6–1* in Sweet Avalanche were significantly down-regulated, while those of Wangxifeng first decreased and then increased. This indicateed that these three genes play different roles in the adaptive regulation of salt stress in the two varieties.

### Analysis of HSP90s protein structure and active sites

The secondary structure of HSP90 protein in rose and raspberry samples is shown in Table 4. In the secondary structure, the alpha helix comprises the largest proportion, ranging from 46.48% to 55.19%, followed by a random coil at approximately 30%. Beta turn has the lowest proportion, ranging from 3.80 to 5.59%. The average proportion of random coils in the first category (Class 1a and 1b) is 28.93%, while the average proportion of proteins in the second category is relatively high, reaching 30.96%. Correspondingly, the alpha helix in the first category accounts for 52.59%, higher than the 50.49% in the second category (Class 2a and 2b). The number of beta turns for Class 1a, 1b, and 2b were all 19, while Class 2a has 21 (except for RcHSP90-2–1). Except for RiHSP90-2–1, there were 25 alpha helices in Class 1a, 24 in Class 1b, 24–29 in Class 2a, and 32–35 in Class 2b. Compared with the first type of HSP90 protein in roses and raspberries, the quantity and proportion of the second type of alpha helix were higher.

**Table 4:** Secondary structure analysis of HSP90 members in roses and raspberries

Name	Class	Alpha helix			Beta turn			Random coil		Extended strand	
		Amino acids	Proportion %	Number	Amino acids	Proportion %	Number	Amino acids	Proportion %	Amino acids	Proportion %
RcHSP90-1–1	1a	380	54.05	25	38	5.41	19	189	26.88	96	13.66
RcHSP90-2–1	2a	390	46.48	27	35	4.17	19	291	34.68	123	14.66
RcHSP90-4–1	2a	414	52.21	27	36	4.54	21	230	29.00	113	14.25
RcHSP90-5–1	1b	380	54.52	24	38	5.45	19	182	26.11	97	13.92
RcHSP90-6–1	1a	365	52.07	25	35	4.99	19	204	29.10	97	13.84
RcHSP90-6–2	2b	419	51.66	32	41	5.06	19	244	30.09	107	13.19
RiHSP90-2–1	1a	239	53.47	16	17	3.80	11	142	31.77	49	10.96
RiHSP90-2–2	2b	452	55.19	35	42	5.13	19	213	26.01	112	13.68
RiHSP90-3–1	1b	358	51.36	24	35	5.02	19	202	28.98	102	14.63
RiHSP90-4–1	2a	401	50.38	24	35	4.40	21	254	31.91	106	13.32
RiHSP90-5–1	Unclassified	354	50.28	26	35	4.97	19	214	30.40	101	14.35
RiHSP90-6–1	2a	377	47.01	29	43	5.36	21	273	34.04	109	13.59
RiHSP90-7–1	1a	358	51.22	25	36	5.15	19	209	29.90	96	13.73
RiHSP90-7–2	1a	356	51.00	25	39	5.59	19	208	29.80	95	13.61

**Fig. 8:**
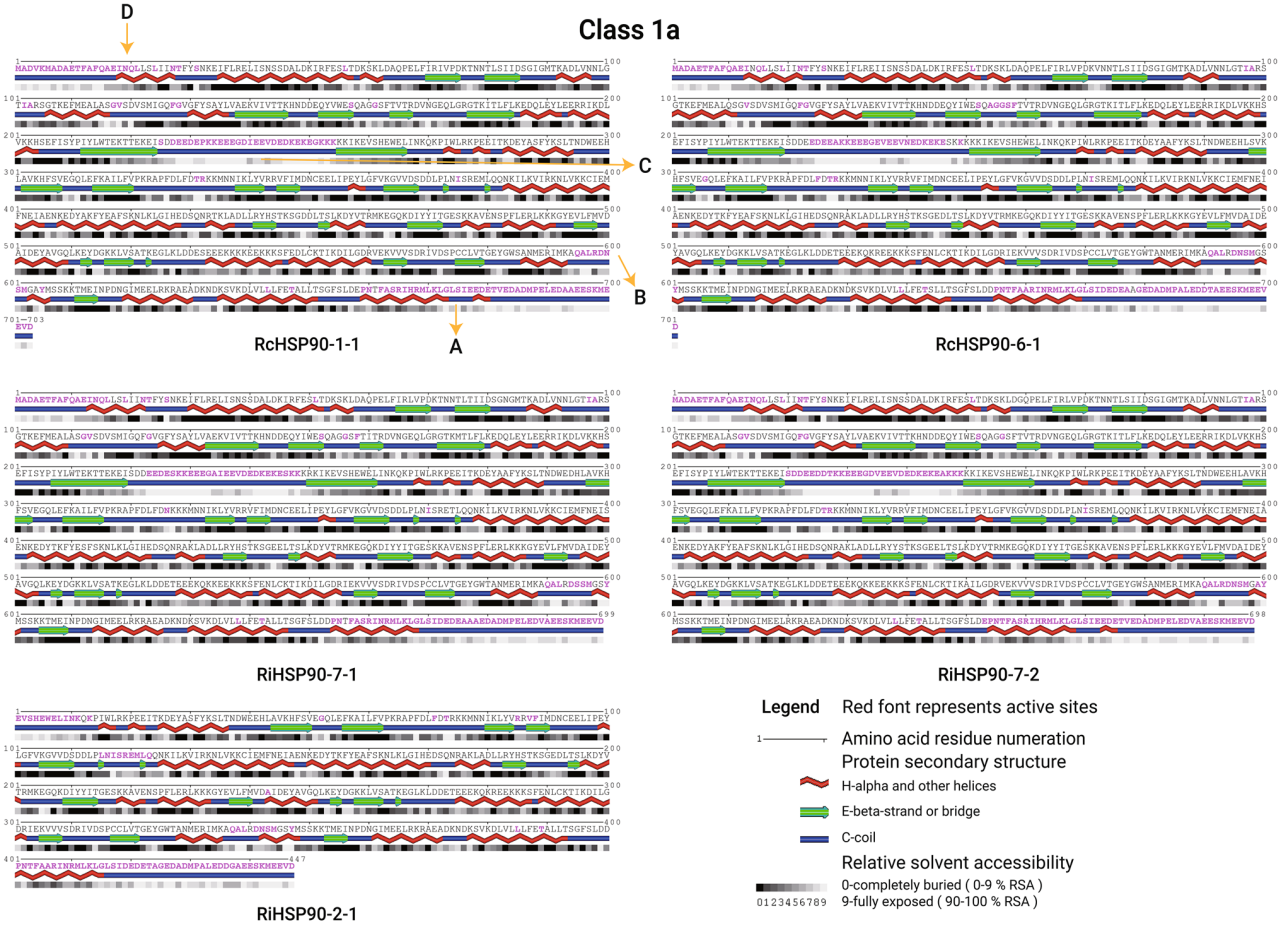
Protein structure and active sites of Class 1a. Purple amino acid markers represent protein active sites

The active sites of HSP90 protein are shown in Fig. 8, S2–S4. Including the proteins of roses and raspberries, the structure of HSP90s in the same subgroup is similar, while the protein structure between different subgroups is slightly different. These findings indicated that the structural differences of homologous genes in HSP90 during evolution were smaller than those between HSP90 proteins in the same family species (roses and raspberries). The active sites of HSP90s were mainly concentrated in four parts (Fig. 8A–D), three were concentrated in the alpha helix and its surroundings (Figs. 8A, B, D), and one was concentrated on the loop (Fig. 8C). The active sites of the second type of HSP90 (Class 2a and 2b, Figures S3-S4) were concentrated in the longer N-terminal extension chain and alpha helix, while the N-terminal extension chain of the first type (Class 1a and 1b, Figs. 8 and S2) is shorter and does not contain alpha helix. The N-terminal of Class 2b protein contains two alpha helices with approximately 15 amino acids; the second one does not feature the distribution of an active site, while the alpha helices of Class 2a were shorter, which is the main difference in the structure and active sites of these two sub histones. The collective findings reveal differences in protein structure and active sites among the subgroups of roses and raspberries to a certain extent, although the similarity among members within the same subgroup is relatively high.

## Discussion

### Different replication modes of *RcHSP90s *and *RiHSP90s* may be the reason for the difference in their quantity

As an important economic ornamental flower globally, the rose accounts for more than 30% of the total global flower trade. The plant is subject to a variety of stresses during production and transportation to the market, which affect its yield and quality; salt and drought are important threats (Liu et al. 2023b; Zhao et al. 2023). The important functions of *Hsp90* in plant growth and development regulation, stress response, and other life activities have been studied in many plants, but there are few studies in rose and raspberry. Whole genome identification has identified six *Hsp90* family members in rose and eight in raspberry, similar to the findings of seven in *Arabidopsis* (Krishna & Gloor 2001) and seven in tomato (Zai et al. 2015); less than the 35 in *Brassica napus* (Wang et al. 2022b), 21 in tobacco (Song et al. 2019), 12 in cabbage (Sajad et al. 2022), and 10 in pumpkin (Hu et al. 2023b); and more than the four in *Dendrobium officinale* (Wang et al. 2022a). *RcHsp90s* and *RiHsp90s* were divided into two major categories and four subgroups (Fig. 1). The amino acid length, isoelectric point, and instability index of Hsp90 members in different subgroups of roses and raspberries were different. The unstable proteins all belong to Class 1a and 2a subgroups (Table 2), indicating that the physicochemical properties of members in each subgroup are also different.

Macro-collinearity analysis showed that the number of *Hsp90* collinearity genes between roses and raspberries, roses and *Arabidopsis*, and roses and rice decreased sequentially, with 6, 3, and 0 genes, respectively (Fig. 3). Both roses and raspberries belong to the Rosaceae family, while they belong to the dicotyledonous family, which is consistent with their close relationship. There are six *RcHSP90s*, with collinearity with *HSP90* in raspberries of also six. There are eight *RiHSP90s*, among them, *RcHSPs* do not have tandem duplication genes or segmental duplication genes, while *RiHSP90-7–1* and *RiHSP90-7–2* are tandem duplication genes, and *RiHSP90-5–1* and *RiHSP90-7–1* are segmental duplication genes (Fig. 2). In other plants, Csa1G569270/Csa1G569290 in cucumber *HSP90* are tandem duplication genes, and no segmental duplication genes (Zhang et al. 2021). *B. napus HSP90* replication includes, proximal, dispersed, and whole genome duplication (Wang et al. 2022b). Pumpkin *HSP90* contains four pairs of segmental duplication, and no tandem duplication (Hu et al. 2023b). *PtHsp90-5a*/*PtHsp90-5b* is the only pair of segmental duplication genes in *Populus* (Zhang et al. 2013). During the evolution of plant genome and genetic systems, gene duplications have been one of the leading causes of the expansion of gene families (Cannon et al. 2004; Hu et al. 2022, 2023a). The amplification of the *HSP90* gene family in each species was performed in a specific way (Zhang et al. 2021), which may be an important reason for the difference in the number of *HSP90s* between roses and raspberries.

### Different protein structures, active sites, and specific expression of HSPs lead to diverse biological functions

Salt stress is one of the most severe environmental factors that restrict crop production globally (Yokoi et al. 2002), and drought has a significant impact on the growth and quality of roses (Su et al. 2023; Zhao et al. 2023; Geng et al. 2023). *RcHSP90s* contains abundant abiotic stress, hormone, and photolight-response acting elements (Fig. 5). The qRT-PCR results of Sweet Avalanche and Wangxifeng (Fig. 7) showed a decreasing trend of the expression levels of *HSP90* genes in Class 1a and 1b subgroups showed under drought and salt stress; the difference became significant, while the expression levels of *RcHSP90-6–2* in Class 2b increased significantly. In summary, *RcHSP90-1–1*, *RcHSP90-5–1*, and *RcHSP90-6–1* in the first category may play important roles in the response of rose to abiotic stresses, such as salt and drought. At the same time, the changing trends of the three genes in the two varieties of Sweet Avalanche and Wangxifeng were not similar, this indicated that the three genes play different roles in the adaptive regulation of drought and salt stress in the two varieties. Previous studies have shown that stiff, leathery leaves are widespread in species adapted to drought occurring in various environments throughout the world, and that waxy coriaceous leaves may help minimize water loss during high water-stress conditions (De Micco and Aronne 2012; Medeiros et al. 2017; Wright et al. 2021), this may be one of the reasons for the differential gene expression between two different leaf type varieties, we will also conduct further research on their differences. *HSP90.2* (*AT5G56010*) and *HSP90.3* in Class 1b play important roles in the response to low temperature stress (Bao et al. 2014). The expressions of *PtHsp90-1a*, *PtHsp90-1b*, and *PtHsp90-3* in the first class of poplar Hsp90 were upregulated under almost all drought stresses, and *PtHsp90-1a* and *PtHsp90-1b* were immediately and highly induced under high temperature conditions (Zhang et al. 2013). In general, different sequences encode different proteins and ultimately perform different functions, while homologous genes have similar gene structures and functions, and sequences with high similarity often play similar roles in different species (Chen et al. 2007). Homologous genes may change in the course of evolution due to changes in environment or selection pressure, and intron gain and/or intron loss, and intron densities were proposed to play an important role in the evolution of large eukaryote genomes (Jeffares et al. 2006). Motif composition and gene structure of *Hsp90* genes in the same subgroup of *Dendrobium officinale, B. napus*, and cucumber are highly similar, with differences between different subgroups (Zhang et al. 2021; Wang et al. 2022a, 2022b). In the present study, the motif composition, gene structure, and protein structure of HSP90 within each subgroup of roses were highly similar, with differences between subgroups (Figs. 2, 8, S2–S4). However, compared to the first type, the second type of protein has a longer random coil in the N segment, and these irregular curls were distributed with a large number of active sites. Compared with Class 2a, the HSP90 protein of Class 2b also features two alpha helices with a length of approximately 15 amino acids, although no active sites were distributed on the second helix. Based on this, the basic structure and active sites of HSP90 protein ensure the consistency of protein basic functions, such as abiotic stress. The difference in N-terminal structure and active sites may be one of the reasons for the differentiation of protein functions.

In summary, the HSP90 members of roses and raspberries, both belonging to the Rosaceae family, have greater similarities. Different replication modes are the reason for the quantity difference between the two proteins. The same subgroup features a similar structure. Differences in motif composition, protein structure, and active sites among different subgroups may be the causes of functional differentiation. *RcHSP90-1–1*, *RcHSP90-5–1*, and *RcHSP90-6–1* may play important roles in the salinity and drought stress of rose These results provide theoretical support for studies of the evolution and function of the *HSP90* family of genes and in breeding rose varieties with strong stress resistance.

## Supplementary Information

Below is the link to the electronic supplementary material.

Supplementary file1 (XLSX 27 KB)

Supplementary file2 Figure S1 Potted rose and experimental treatments. Left: potted rose, 4-6 cuttings per pot; Medium: distilled water was the solution of the control group; Right treatment group: 10% PEG6000 or 200mM NaCl solution (PDF 1589 KB)

Supplementary file3 Figure S2 Protein structure and active sites of Class 1b (PDF 1591 KB)

Supplementary file4 Figure S3 Protein structure and active sites of Class 2a (PDF 1240 KB)

Supplementary file5 Figure S4 Protein structure and active sites of Class 2b (PDF 1178 KB)

Supplementary file6 (DOCX 16 KB)

Supplementary file7 (XLSX 11 KB)

## Data Availability

All relevant data are within the manuscript and its Additional files.
