# Supplementary figures and images for: Genome-wide identification of HSP90 gene family in Rosa chinensis and its response to salt and drought stresses
Source: 3 Biotech. 2024 Aug 18;14(9):204. doi: 10.1007/s13205-024-04052-0 (PMC11330952; doi:10.1007/s13205-024-04052-0)

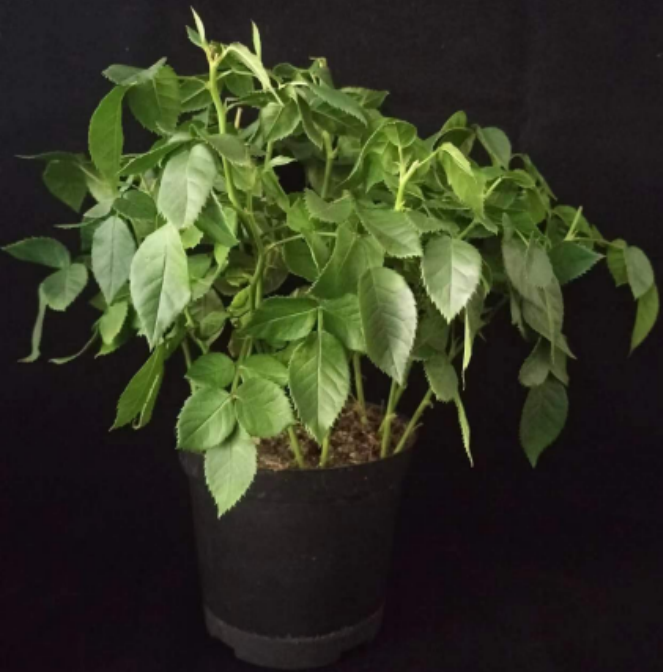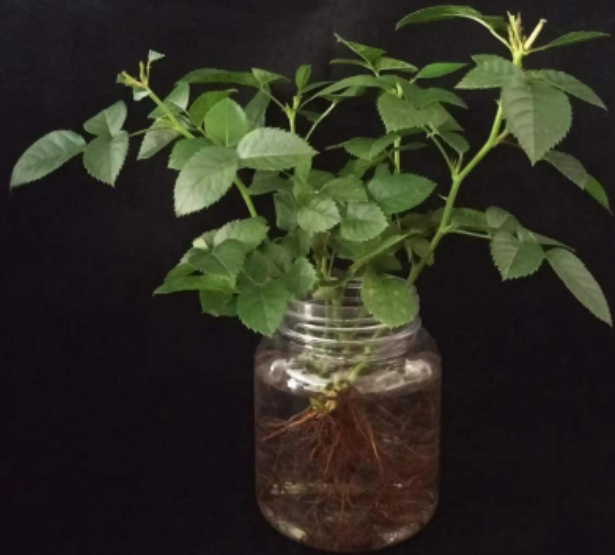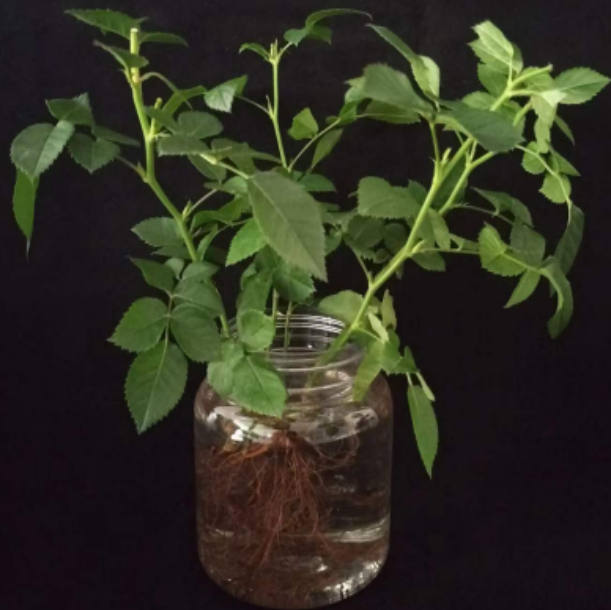

Supplement: Supplementary file 2 — Supplementary file2 Figure S1 Potted rose and experimental treatments. Left: potted rose, 4-6 cuttings per pot; Medium: distilled water was the solution of the control group; Right treatment group: 10% PEG6000 or 200mM NaCl solution (PDF 1589 KB) [file 13205_2024_4052_MOESM2_ESM.pdf]
