# Supplementary material for: Genome-wide identification of HSP90 gene family in Rosa chinensis and its response to salt and drought stresses
Source: 3 Biotech. 2024 Aug 18;14(9):204. doi: 10.1007/s13205-024-04052-0 (PMC11330952; doi:10.1007/s13205-024-04052-0)
Supplement: Supplementary file 3 — Supplementary file3 Figure S2 Protein structure and active sites of Class 1b (PDF 1591 KB) [file 13205_2024_4052_MOESM3_ESM.pdf]

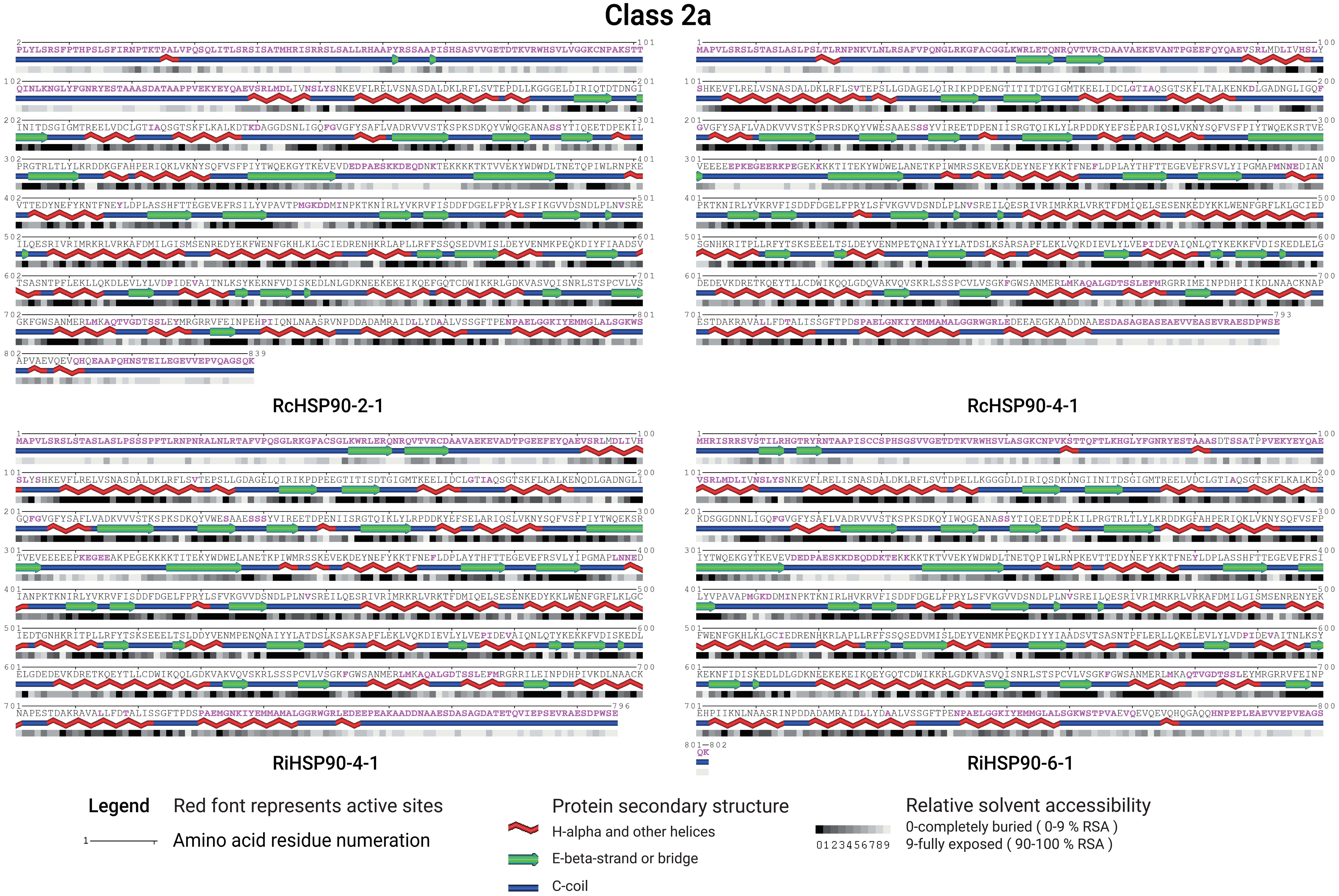
Class 2a
RcHSP90-2-1
RcHSP90-4-1
RiHSP90-4-1
RiHSP90-6-1
Legend
Red font represents active sites
Amino acid residue numeration
Protein secondary structure
H-alpha and other helices
E-beta-strand or bridge
C-coil
Relative solvent accessibility
0-completely buried (0-9 % RSA)
9-fully exposed (90-100 % RSA)
0123456789
