# Supplementary material for: Genome-wide identification of HSP90 gene family in Rosa chinensis and its response to salt and drought stresses
Source: 3 Biotech. 2024 Aug 18;14(9):204. doi: 10.1007/s13205-024-04052-0 (PMC11330952; doi:10.1007/s13205-024-04052-0)
Supplement: Supplementary file 4 — Supplementary file4 Figure S3 Protein structure and active sites of Class 2a (PDF 1240 KB) [file 13205_2024_4052_MOESM4_ESM.pdf]

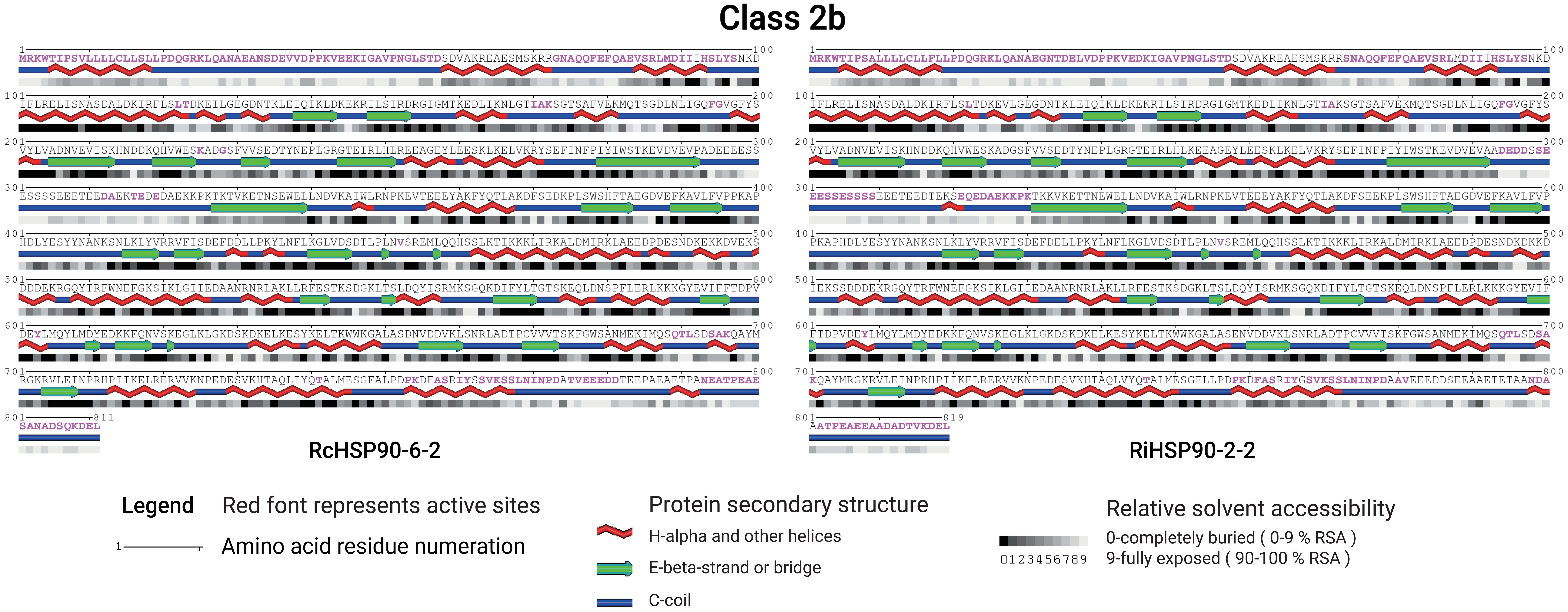

Class 2b
RcHSP90-6-2
RiHSP90-2-2
Legend
Red font represents active sites
Amino acid residue numeration
Protein secondary structure
H-alpha and other helices
E-beta-strand or bridge
C-coil
Relative solvent accessibility
0123456789
0-completely buried (0-9 % RSA)
9-fully exposed (90-100 % RSA)
