# Supplementary material for: Genome-wide identification of HSP90 gene family in Rosa chinensis and its response to salt and drought stresses
Source: 3 Biotech. 2024 Aug 18;14(9):204. doi: 10.1007/s13205-024-04052-0 (PMC11330952; doi:10.1007/s13205-024-04052-0)
Supplement: Supplementary file 6 — Supplementary file6 (DOCX 16 KB) [file 13205_2024_4052_MOESM6_ESM.docx]

**Table S1 Primer sequences for qRT-PCR**

| Name | Sequence (5’-3’)-F | Sequence (5’-3’)-R |
| --- | --- | --- |
| *RcTCTP* | GGGTGATGATGCAGCTTT | TTAGCACTTGACCTCCTTCA |
| *RcHSP90-1-1* | GCTGACCTTCTCCGATACCA | CACTGCCTTCTTGCTCTCAC |
| *RcHSP90-2-1* | GGTCAGTTTGGTGTTGGCTT | TTGCCTCTCCTTGCCATACA |
| *RcHSP90-5-1* | CGATTCTCCCTGCTGTTTGG | TGCATCAGCCCTCTTTCTGA |
| *RcHSP90-6-1* | TGTGGATTCGCCTTGCTGCT | TCCCTGAGAGCCTGAGCCTT |
| *RcHSP90-6-2* | AGGCACCCAATCATCAAGGA | AGAGCAAAGCCACTCTCCAT |
